# Bis-Enoxacin Blocks Rat Alveolar Bone Resorption from Experimental Periodontitis

**DOI:** 10.1371/journal.pone.0092119

**Published:** 2014-03-17

**Authors:** Mercedes F. Rivera, Sasanka S. Chukkapalli, Irina M. Velsko, Ju-Youn Lee, Indraneel Bhattacharyya, Calogero Dolce, Edgardo J. Toro, L. Shannon Holliday, Lakshmyya Kesavalu

**Affiliations:** 1 Department of Periodontology, College of Dentistry, University of Florida, Gainesville, Florida, United States of America; 2 Department of Oral Biology, College of Dentistry, University of Florida, Gainesville, Florida, United States of America; 3 Department of Oral Diagnostic Sciences, College of Dentistry, University of Florida, Gainesville, Florida, United States of America; 4 Department of Orthodontics, College of Dentistry, University of Florida, Gainesville, Florida, United States of America; 5 Department of Anatomy and Cell Biology, College of Medicine, University of Florida, Gainesville, Florida, United States of America; University of Toronto, Canada

## Abstract

Periodontal diseases are multifactorial, caused by polymicrobial subgingival pathogens, including *Porphyromonas gingivalis*, *Treponema denticola*, and *Tannerella forsythia*. Chronic periodontal infection results in inflammation, destruction of connective tissues, periodontal ligament, and alveolar bone resorption, and ultimately tooth loss. Enoxacin and a bisphosphonate derivative of enoxacin (bis-enoxacin) inhibit osteoclast formation and bone resorption and also contain antibiotic properties. Our study proposes that enoxacin and/or bis-enoxacin may be useful in reducing alveolar bone resorption and possibly bacterial colonization. Rats were infected with 10^9^ cells of polymicrobial inoculum consisting of *P. gingivalis*, *T. denticola*, and *T. forsythia*, as an oral lavage every other week for twelve weeks. Daily subcutaneous injections of enoxacin (5 mg/kg/day), bis-enoxacin (5, 25 mg/kg/day), alendronate (1, 10 mg/kg/day), or doxycycline (5 mg/day) were administered after 6 weeks of polymicrobial infection. Periodontal disease parameters, including bacterial colonization/infection, immune response, inflammation, alveolar bone resorption, and systemic spread, were assessed post-euthanasia. All three periodontal pathogens colonized the rat oral cavity during polymicrobial infection. Polymicrobial infection induced an increase in total alveolar bone resorption, intrabony defects, and gingival inflammation. Treatment with bis-enoxacin significantly decreased alveolar bone resorption more effectively than either alendronate or doxycycline. Histologic examination revealed that treatment with bis-enoxacin and enoxacin reduced gingival inflammation and decreased apical migration of junctional epithelium. These data support the hypothesis that bis-enoxacin and enoxacin may be useful for the treatment of periodontal disease.

## Introduction

Periodontal disease (PD) is a polymicrobial subgingival biofilm-mediated inflammatory disease of the periodontal tissues (periodontal ligament, connective tissue, alveolar bone) surrounding and supporting the teeth [1], [2]. An estimated 5–20% of the world's population suffers from generalized periodontitis; it is among the most common chronic infections in humans [3]. Periodontitis is now recognized as a risk and contributing factor to systemic diseases, including atherosclerotic vascular disease [4], [5], diabetes [6], rheumatoid arthritis [7], Alzheimer's disease [8], [9], and respiratory disease [10]. A chronic immune and inflammatory response triggered by periodontal bacteria, *Porphyromonas gingivalis*, *Treponema denticola* and *Tannerella forsythia*, which are strongly associated with periodontal disease in humans and are together known as the “red complex”, results in severe destruction of the periodontium [1], [2]. Classical periodontal disease characteristics include apical migration of the junctional epithelium (JE), gingival ulcerations, formation of periodontal pockets at the gingival sulcus, and alveolar bone resorption resulting in tooth mobility and eventually tooth loss.

The most destructive effects of PD result from alveolar bone resorption caused by the activation of osteoclasts as a result of inflammation [11]. This is known to be dependent on osteoclasts, as bone resorption was shown to be absent in periodontally-infected transgenic rats that lack osteoclasts [12]. It is therefore plausible that anti-resorptive agents could be employed in patients with periodontal infection to prevent alveolar bone resorption [13]. However, because current therapeutic anti-resorptives have been linked to oral osteonecrosis, the use of current anti-resorptives to block alveolar bone resorption associated with periodontal bacterial pathogens has an unacceptably high risk of oral osteonecrosis, a severe and debilitating condition [14], [15]. The precise mechanism by which current anti-resorptives [nitrogen-containing bisphosphonates like Fosamax (alendronate) and denosumab (a receptor activator of nuclear factor-κB-ligand (RANKL) antibody)] provoke oral osteonecrosis is unknown [16]. It is possible to argue that anti-resorptives that inhibit osteoclasts by a different mechanism from current anti-resorptives will not predispose toward oral osteonecrosis.

Enoxacin, a fluoroquinolone antibiotic and anti-resorptive agent, inhibits osteoclast formation and bone resorption by inhibiting binding between the B-subunit of vacuolar H+-ATPase (V-ATPase) and microfilaments [17], [18]. Previous findings show that interaction between vacuolar H^+^-ATPase (V-ATPase) and microfilaments is vital for osteoclast function [19], [20]. Enoxacin has also been reported to have the ability to stimulate microRNA activity and this has been shown to block cancer growth and metastasis [21], [22]. Bis-enoxacin is a bisphosphonate derivative of enoxacin. Previously bis-enoxacin was shown to bind bone and retain the antibiotic activity of its parent molecule [23]. More recent studies show that bis-enoxacin blocks binding between recombinant B-subunit and microfilaments, inhibits osteoclastogenesis and bone resorption in cell culture, and inhibits aseptic, mechanically-induced resorption *in vivo* [24].

In this paper we have tested the effects of enoxacin and bis-enoxacin in a rat model of polymicrobial periodontal disease. We have compared these molecules with a pure anti-resorptive, alendronate, which is a widely used and well characterized nitrogen-containing bisphosphonate that inhibits resorption by a different mechanism from enoxacin and bis-enoxacin [24], [25]. In addition, we have used doxycycline as an antibiotic control; we find that bis-enoxacin functions as well or better than both alendronate and doxycycline. These data provide proof-of-principle for the use of bis-enoxacin as a therapeutic agent for the treatment of periodontal disease and suggests the need for further studies to evaluate the efficacy and safety of bis-enoxacin.

## Materials and Methods

### Bacterial strains and inoculum preparation

*P. gingivalis* FDC 381, *T. denticola* ATCC 35404, and *T. forsythia* ATCC 43037 were grown anaerobically at 37°C as described previously [26]–[29]. Cell concentrations for each species were determined and cells were suspended in reduced transport fluid. *P. gingivalis* was mixed with an equal cell quantity of *T. denticola*, vortexes, and left to sit for 5 min; subsequently, an equal cell quantity of *T. forsythia* was added to the culture tubes containing *P. gingivalis* and *T. denticola*, and cells were vortexes and allowed to interact for an additional 5 min. The suspension was then mixed with an equal volume of 8% sterile carboxymethylcellulose (CMC) (Sigma-Aldrich, St. Louis, MO) in phosphate buffered saline (PBS) [27], [29], [30].

### Oral infection and oral sampling

Female Sprague-Dawley rats were received from Harlan Laboratories (Harlan, Indianapolis, IN) and fed powdered normal chow (Harlan; Teklad Global Diet, Indianapolis, IN) and water *ad libitum*. This study was carried out in strict accordance with the recommendations in the Guide for the Care and Use of Laboratory Animals of the National Institutes of Health. The protocol was approved by the Institutional Animal Care and Use Committee of the University of Florida (Protocol # 201004367). The University of Florida has an Assurance with OLAW and follows PHS policy, the Animal Welfare Act and Animal Welfare Regulations, and the Guide for the Care and Use of Laboratory Animals. The University of Florida is also AAALAC accredited. Rats were administered an antibiotic via drinking water, followed by oral swabbing with 0.12% chlorhexidine gluconate mouth rinse as described in [27], [29], [30] to inhibit endogenous microorganisms in the rat oral cavity and to enhance subsequent colonization of periodontal bacteria. A polymicrobial inocula of 1×10^9^ cells was administered as oral lavage every other week for 12 weeks [27], [29], [30] to establish a stable infection. Sham-infected control rats (Gr VIII) received sterile 4% CMC only. Post-infection oral samples were collected after every infection cycle via swabbing of the oral cavity using a 2.6 mm thick cotton tip (VWR, Radnor, PA).

### Treatment groups

Rats were randomly divided into eight groups (n = 6): Group I polymicrobial infection with *P. gingivalis*/*T. denticola/T. forsythia* only; II polymicrobial infection plus treatment with bis-enoxacin (5 mg/kg/day) [24]; III polymicrobial infection plus treatment with bis-enoxacin (25 mg/kg/day) [24]; IV polymicrobial infection plus treatment with alendronate (1 mg/kg/day) [31]; V polymicrobial infection plus treatment with alendronate (10 mg/kg/day) [31]; VI polymicrobial infection plus treatment with enoxacin (5 mg/kg/day) [24], [25]; VII polymicrobial infection plus treatment with doxycycline (5 mg/day) [32]; and VIII sham-infected, untreated controls. Treatments were given daily for 6 weeks following 6 weeks of infections as described above. Injections were given subcutaneously at the base of the neck while the rats were anesthetized by isoflurane inhalation. The lower dose of 5 mg/kg mixture of bis-enoxacin powder was suspended in sterile PBS, whereas the higher dose of 25 mg/kg was more difficult to dilute and therefore was suspended in 5% ethanol. Enoxacin (5 mg/kg) mixture powder was suspended in 0.1 M NaOH. Both 1 mg/kg and 10 mg/kg alendronate mixtures and the 5 mg/day doxycycline mixture were suspended in sterile PBS.

### Detection of bacterial genomic DNA in oral samples

DNA was isolated from rat oral samples using the Wizard Genomic DNA Purification Kit (Promega, Madison, WI), following manufacturer's protocol [27]. PCR was performed using 16S rRNA gene species-specific PCR oligonucleotide primers (*P. gingivalis*): 5′-TGTAGATGACTGATGGTGAAAACC-3′ (forward), 5′-ACGTCATCCCCACCTTCCTC-3′ (reverse); (*T. denticola*) 5′-TAATACCGAATGTGCTCATTTACAT-3′(forward), 5′-CTGCCATATCTCTATGTCATTGCTCTT-3′ (reverse); and (*T. forsythia*) 5′-AAAACAGGGGTTCCGCATGG-3′ (forward), 5′-TTCACCGCGGACTTAACAGC-3′ (reverse), resulting in band sizes of 600 bp, 860 bp, and 426 bp, respectively. Genomic DNA extracted individually from all three bacteria served as positive PCR controls and PCR performed with no template DNA served as negative PCR controls. Each PCR assay was detectable at 0.05 pg of DNA standard.

### Detection of bacterial genomic DNA in internal organs

Following 12 weeks of polymicrobial infections with an overlapping 6 weeks of treatment, rats were euthanized and aorta, heart, liver, and spleen were collected. DNA was isolated from 25 mg of each of the tissues using the DNeasy Blood and Tissue Kit (Qiagen, Valencia, CA.) for DNA purification with no deviation from manufacturer's protocol [27], [29]. PCR was performed as described in the previous section.

### Polymicrobial infection-induced IgG and IgM antibody analysis

Prior to euthanasia, blood was collected and sera were stored at -20°C for immunoglobulin G (IgG) and immunoglobulin M (IgM) antibody analysis. Sera were used to determine IgG or IgM antibody concentrations against whole cells of *P. gingivalis, T. denticola*, and *T. forsythia* using a standard ELISA protocol as described in [26], [28], [33]. Whole *P. gingivalis, T. denticola*, and *T. forsythia* cells were treated overnight with 0.5% formalin in buffered saline, washed, diluted to OD_600_ 0.3, and coated in wells of microtiter plates. Sera were diluted (1:100 for IgG and 1:20 for IgM) and were reacted with the bacterial antigen for 2 hours at room temperature. The secondary antibody goat anti-rat IgG and IgM, conjugated to alkaline phosphatase (1:5000) (Bethyl Laboratories, Montgomery, TX), was added to the plates and the assay developed with *p*-nitrophenolphosphate (Sigma-Aldrich). The assay reactions were terminated by the addition of 3 M NaOH and analyzed at OD_405_ using a Bio-Rad Microplate Reader. Rat serum antibody concentrations were assessed using a gravimetric standard curve, detected and developed as described [26], [28].

### Histological analysis of right mandible

The right mandible was collected post euthanasia and suspended in 10% buffered formalin and decalcified using decalcifying solution (Richard-Allan Scientific, Kalamazoo, MI) for 2 weeks. Five μm thick sections were cut and stained with hematoxylin and eosin (H&E) for histology and histomorphometric analysis [26], [28], [33]. The interdental area was examined for increase in inflammation, apical migration of JE and proliferation, and vertical bone resorption in a blinded manner. This interdental region between the alveolar bone crest (ABC) and the cemento-enamel junction (CEJ) is where bone resorption and inflammatory process is typically assessed [33]. Three 50 mm^2^ areas were examined for inflammatory cells and the counts were averaged. The linear distance between the ABC and the CEJ was measured to determine vertical alveolar bone resorption.

### Alveolar bone resorption and intrabony defects

Both the left maxilla and mandible were removed, autoclaved, defleshed and immersed in 3% hydrogen peroxide overnight. Prior to staining, the maxillae and mandibles were tilted and stabilized with dental wax under a 10 x stereo dissecting microscope (SteReo Discovery V8) to verify the presence of the intrabony defects in buccal and lingual surfaces. The pattern of vertical alveolar bone resorption was measured after staining with 0.1% methylene blue solution to delineate cemento-enamel junction [26], [28], [33]. Digital images of buccal and lingual root surfaces of all molar teeth were captured under a 10 x stereo dissecting microscope (SteReo Discovery V8; Carl Zeiss Microimaging Inc, Thornwood, NY). The surface perimeters of CEJ and ABC were traced using the calibrated line tool (AxioVision LE 29A software version 4.6.3.). Two blinded examiners performed all measurements. The means of the measurements were obtained for two of the four quadrants in the mouth.

### Statistical analysis

A power analysis was performed with respect to the simple comparison of the calvarial bone osteoclast numbers/mm^2^ bone area using our published data [34]. A two-tailed t-test with significance of 0.05 resulted in a power of 0.8 for detecting a mean difference of 14.45 with a pooled standard deviation of 7.14 that was estimated from four groups. Each group contained 5 animals and the pooled average difference between the bacterial challenged groups and the control was 16. Thus, this value supports that a minimum of 5 rats per group should be sufficient to identify statistical differences between the groups. Therefore, rats were randomly divided into eight groups and each group contained 6 rats (n = 6). Antibody analysis and alveolar bone resorption data are presented as means ± standard deviations (SD). An unpaired, two-tailed Student's *t*-test was used to compare two independent groups. For all statistical analysis, Prism for Windows, Version 5.0 (GraphPad Software, San Diego, CA) was used and *P*<0.05 was considered significant.

## Results

### Oral bacterial colonization

The presence of *P. gingivalis, T. denticola*, and *T. forsythia* in oral samples was evaluated sequentially after each infection time point to ensure successful oral colonization and chronic infection. Six samples were collected throughout the study. By the fifth sample 100% of the rats were positive for *P. gingivalis* genomic DNA. One hundred percent of the rats were positive for *T. denticola* genomic DNA by the sixth sample and all of the rats but one was positive for *T. forsythia* genomic DNA by the fifth sample. None of the sham-infected rats were positive for bacterial DNA (Table 1). These results confirmed oral colonization of the inoculated bacteria and the absence of infection in sham-infected rats.

**Table 1:** Distribution of oral plaque samples positive for bacterial DNA by PCR.

		*P. gingivalis*						*T. denticola*						*T. forsythia*					
Groups	n	1^a^	2	3	4	5	6	1	2	3	4	5	6	1	2	3	4	5	6
*Pg+Td+Tf*	6	0^b^	N/P^c^	5	0	4	0	1	0	1	6	5	6	6	0	1	0	0	0
*Pg+Td+Tf* + Bis-enoxacin 5 mg	6	0	N/P^c^	6	0	6	N/P^c^	3	0	3	0	5	4	5	0	N/P^c^	N/P^c^	0	0
*Pg+Td+Tf* + Bis-enoxacin 25 mg	6	0	N/P^c^	5	0	3	N/P^c^	3	2	3	0	5	5	3	0	N/P^c^	N/P^c^	3	N/P^c^
*Pg+Td+Tf* + Alendronate 1 mg	6	0	N/P^c^	6	0	6	N/P^c^	3	1	6	0	5	2	5	1	N/P^c^	N/P^c^	0	N/P^c^
*Pg+Td+Tf* + Alendronate 10 mg	6	0	N/P^c^	5	0	6	N/P^c^	3	2	6	0	5	5	4	0	N/P^c^	N/P^c^	3	N/P^c^
*Pg+Td+Tf* + Enoxacin 5 mg	6	0	N/P^c^	0	1	6	N/P^c^	2	2	6	0	5	5	6	1	N/P^c^	N/P^c^	0	N/P^c^
*Pg+Td+Tf* + Doxycycline 5 mg	6	0	N/P^c^	0	1	5	N/P^c^	5	3	5	0	4	4	4	2	N/P^c^	N/P^c^	0	N/P^c^
Control	6	0	N/P^c^	0	N/P^c^	N/P^c^	N/P^c^	0	0	0	N/P^c^	N/P^c^	0	0	0	0	N/P^c^	N/P^c^	N/P^c^

Rats were infected with *Pg+Td+Tf* (*P. gingivalis + T. denticola* + *T. forsythia*) for four consecutive days, every other week for 12 weeks and oral samples were collected four days following each infection cycle. Oral samples were analyzed using appropriate bacteria specific PCR primers with positive and negative controls. ^a^ Indicates the number of oral samples collected following polymicrobial infection. ^b^ Indicates the total number of rat oral samples positive by PCR analysis. Oral samples from uninfected control rats were also collected and evaluated for the presence of *P*. *gingivalis*, *T. denticola*, and *T. forsythia* bacterial genomic DNA using bacteria-specific primers. ^c^ N/P indicates that PCR was not performed using respective primers due to insufficient quantity of available DNA. Total number of rats analyzed in each group n = 6. The numbers in each column indicate the number of rats whose respective organ was found positive for bacterial genomic DNA for six rats in each group.

### Humoral immune response to oral infection

**Figure 1:**
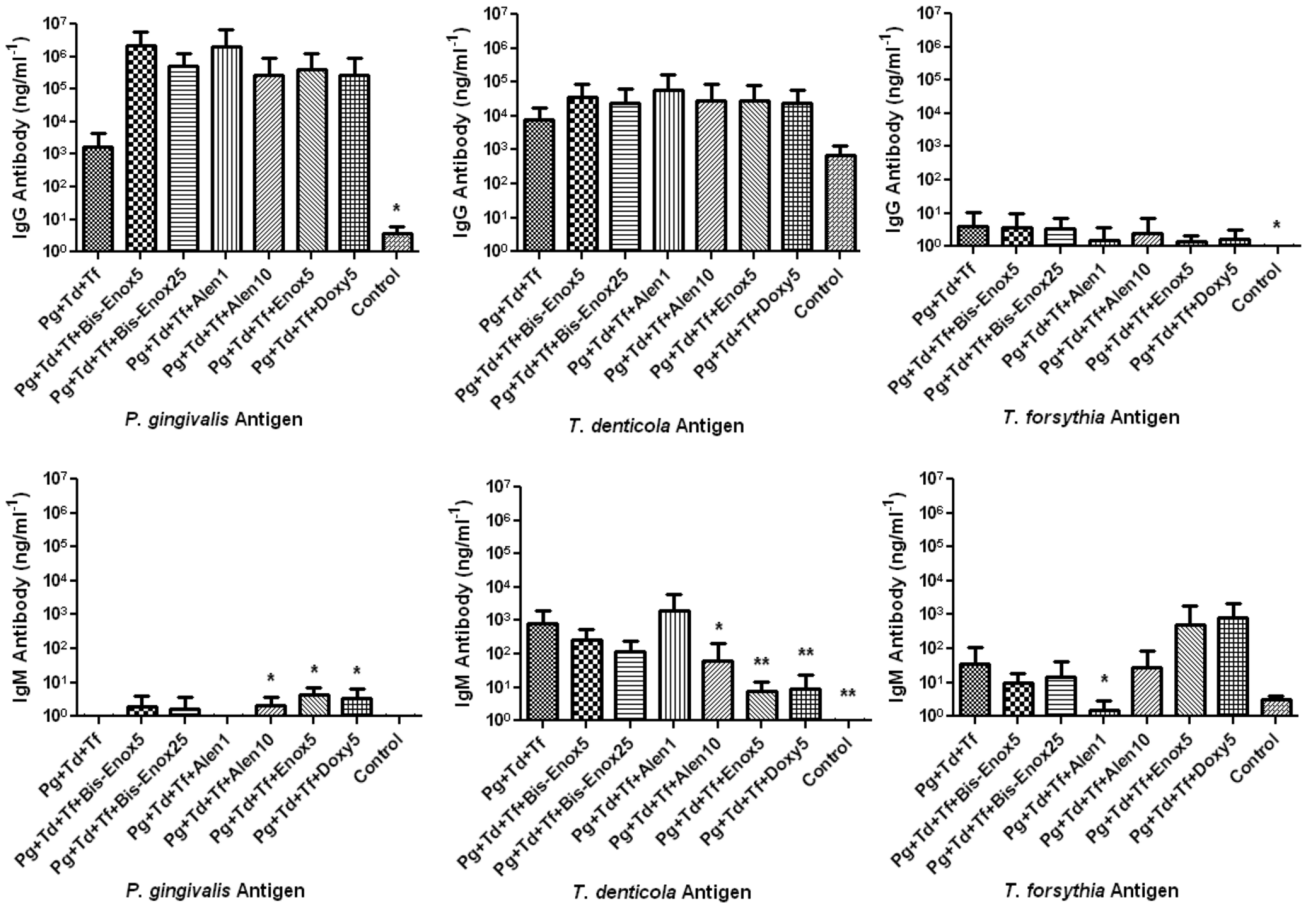
Serum Antibody Response: Serum IgG and IgM antibody levels were measured by ELISA. Serum was collected post-euthanasia at 12 weeks following initial polymicrobial infection (n = 6). Each graph shows the results of serum IgG or IgM antibody levels when probing with either *P. gingivalis, T.denticola* or *T. forsythia* whole cell antigen. Asterisks indicate the following *P*-values when compared to controls *P<0.05; **P<0.005.

As confirmation of an immunological response, sera were evaluated for a humoral response against whole cell antigens of *P. gingivalis, T. denticola*, or *T. forsythia* using an ELISA method. Results show an increase (although not significant) in IgG antibody level when probing with both *P. gingivalis* and *T. denticola* antigen in all treatment groups. No significant increase in IgG antibody level was seen when probing with *T. forsythia* antigen for any group (Figure 1). IgM antibody level for the alendronate (10 mg/kg), enoxacin (5 mg/kg) and doxycycline (5 mg/kg) groups were all significantly increased (*P*<0.05) when probing for *P. gingivalis*. IgM antibody level in the alendronate (10 mg/kg), enoxacin (5 mg/kg) and doxycycline (5 mg/kg) groups were significantly decreased (*P*<0.05 and 0.01) when probing for *T. denticola*, as was the alendronate (1 mg/kg) group when probing for *T. forsythia* (Figure 1).

### Bacteria-induced alveolar bone resorption

**Figure 2:**
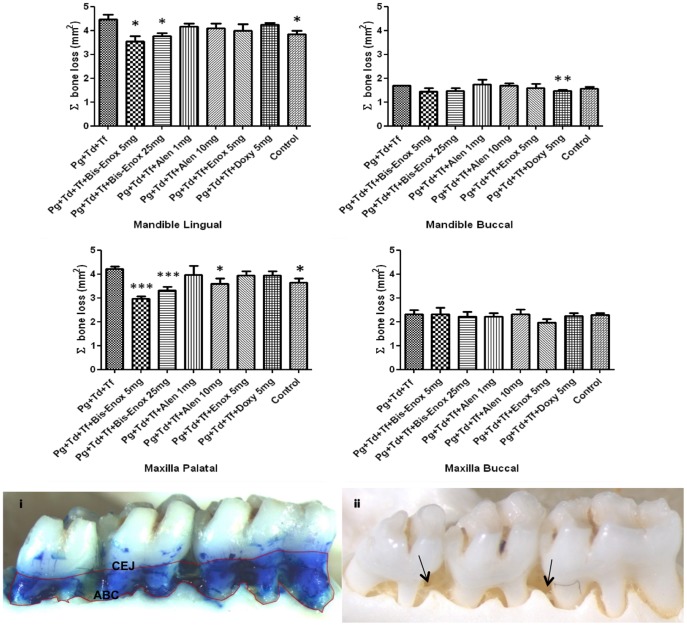
The area of bone resorption was measured in mm^2^. Analysis of the buccal and palatal/lingual surfaces of maxillae and mandibles were done post-euthanasia after 12 weeks of infection, including an overlapping 6 weeks of treatment. All groups were compared to *Pg+Td+Tf* (*P. gingivalis + T. denticola* + *T. forsythia*) group for statistical significance. Asterisks indicate the following *P*-values when compared to controls *P<0.05; **P<0.005; ***P<0.0001. Values are means ± standard deviations of the bone resorption measured. (i) Representative image of the area between the cemento-enamel junction (CEJ) and the alveolar bone crest (ABC) that is measured for bone resorption. (ii) Representative image with black arrows showing how intrabony defects were identified.

The progression of periodontal disease resulting from polymicrobial infection with *P. gingivalis, T. denticola*, and *T. forsythia* and/or treatment was determined by examining alveolar bone resorption and the presence or absence of intrabony defects. Bis-enoxacin (5, 25 mg/kg), alendronate (10 mg/kg) and doxycycline (5 mg/day) treatment groups displayed significant decreases (*P*<0.0001; *P*<0.05; and *P*<0.05, respectively) in maxilla palatal alveolar bone resorption when compared to the infected-untreated group (Table 2; Figure 2). Bis-enoxacin (25 mg/kg), alendronate (1 mg/kg) and doxycycline (5 mg/day) treatments also significantly (*P*<0.05) reduced the percent of intrabony defect (Table 2; Figure 2) consistent with their anti-resorptive properties. No signs of osteonecrosis or inflammation of the bone were observed in histological sections of the jaw specimens using the morphometric methods employed.

**Table 2:** Morphometric measurements of horizontal area alveolar bone resorption and Intrabony defects on the buccal and palatal/lingual surfaces of all molars.

Infection/Groups	Mandible Lingual (mm^2^)	Mandible Buccal (mm^2^)	Maxilla Palatal (mm^2^)	Maxilla Buccal (mm^2^)	Intrabony Defect (%)
*Pg+Td+Tf*	4.463±0.207 n = 6	1.684±0.019 n = 5	4.212±0.112 n = 6	2.317±0.175 n = 5	9.7 n = 6
*Pg+Td+Tf*+Bis-Enoxacin (5 mg/kg)	3.533±0.239 n = 5*	1.447±0.133 n = 5	2.967±0.094 n = 5***	2.326±0.273 n = 5	9.7 n = 6
*Pg+Td+Tf*+Bis-Enoxacin (25 mg/kg)	3.774±0.107 n = 5*	1.463±0.120 n = 5	3.316±0.145 n = 6***	2.209±0.200 n = 5	2.8* n = 6
*Pg+Td+Tf*+Alendronate (1 mg/kg)	4.168±0.131 n = 6	1.750±0.203 n = 6	3.966±0.371 n = 6	2.226±0.145 n = 6	1.4* n = 6
*Pg+Td+Tf*+Alendronate (10 mg/kg)	4.084±0.195 n = 6	1.698±0.101 n = 6	3.586±0.218 n = 5*	2.313±0.207 n = 5	9.7 n = 6
*Pg+Td+Tf*+Enoxacin (5 mg/kg)	3.996±0.263 n = 5	1.591±0.178 n = 5	3.939±0.172 n = 5	1.979±0.148 n = 5	9.7 n = 6
*Pg+Td+Tf*+Doxycyline (5 mg/day)	4.242±0.079 n = 5	1.459±0.048 n = 5**	3.943±0.182 n = 5	2.237±0.142 n = 5	2.8* n = 6
Sham-infected control	3.848±0.142 n = 6*	1.579±0.067 n = 5	3.645±0.161 n = 6*	2.292±0.075 n = 5	5.6 n = 6

Morphometric analysis of the buccal and palatal/lingual surfaces of maxillae and mandibles were done post-euthanasia after 12 weeks of infection, including an overlapping 6 weeks of treatment. The area of total bone resorption was measured in mm^2^. The last column shows the percent intrabony defect found in all jaws. Percentages indicate number of sites found to contain intrabony defects per 72 total sites analyzed per group. All groups were compared to the *Pg+Td+Tf* (*P. gingivalis + T. denticola* + *T. forsythia*) group for statistical significance. Asterisks indicate the following *P*-values when compared to controls *P<0.05; **P<0.005; ***P<0.0001. Values are means ± standard deviations of the bone resorption measured or (n) number of animals. Broken jaws or jaws missing teeth were excluded and reflected in (n).

### Histology of periodontal inflammation

**Figure 3:**
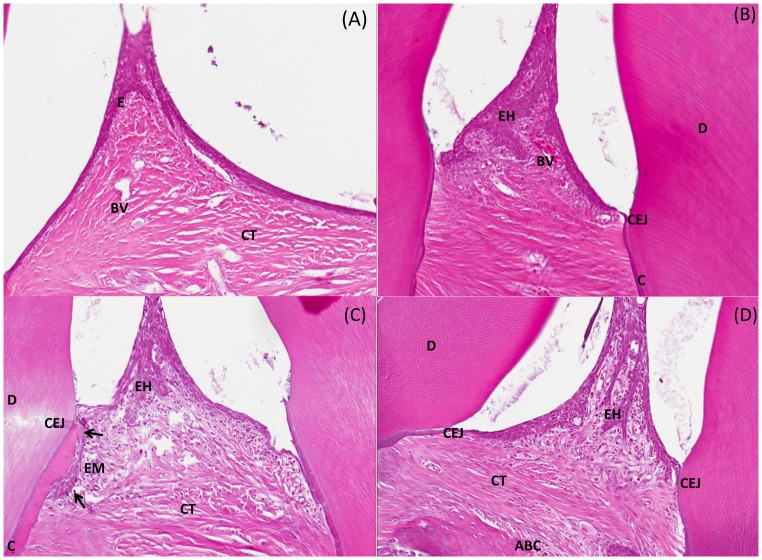
Comparative mandibular histology of alveolar bone sections from maxilla of rat infected with *P. gingivalis/T. denticola/T. forsythia* at 12 weeks. Photomicrograph of the interdental area from rat jaws (all sections stained with hematoxylin and eosin and images captured at 200X magnification). **A**. Section from sham-infected rat displaying minimal inflammation and hyperplasia of the crevicular epithelium (E). **B**. **C**. and **D**. Representative sections from the infected-untreated rats exhibit mild to moderate inflammation and hyperplasia of the epithelium with elongation of the rete ridges (EH) and increase in small capillaries (BV). Apical junctional epithelium migration (EM) is noted in the Section C (black arrows). Dentin (D); Cementum (C); Connective tissue (CT); Cemento-enamel junction (CEJ); Alveolar bone crest (ABC).

To assess bis-enoxacin as an anti-inflammatory agent, gingival inflammation, JE migration, and proliferation were evaluated for the interdental area of sections from the right mandible. Significant decreases (between *P*<0.05 and *P*<0.01) of these parameters were detected with the treatment of bis-enoxacin (25 mg/kg), alendronate (1 and 10 mg/kg) and doxycycline (5 mg/day) when compared to the infected-untreated group (Table 3). When compared to the histology from the control rats (Figure 3A), the histological samples from infected-untreated rats (Figure 3B–D) showed significant (*P*<0.05) increases in inflammation of the interdental epithelium. Elongation of the rete ridges (downward extending epidermal thickening between the dermal papillae caused by periodontal inflammation), leukocytic exocytosis, edema and occasional foci of epithelial migration were also noted in the infected samples. The inflammation was mostly limited to the soft tissue and did not involve subjacent/adjacent alveolar bone.

**Table 3:** Histological analysis of rat periodontal tissue.

Groups	Apical Migration (μm)	Vertical Bone Resorption (μm)	PMN Count (50 μm^2^ area)	Blood Vessel Count (50 μm^2^ area)
*Pg+Td+Tf*	103.6±40.0	407.7±125.5	5.1±3.2	3.2±2.8
*Pg+Td+Tf* + Bis-enoxacin 5 mg	86.1±7.8	284.9±99.5	3.0±1.8	1.5±1.8
*Pg+Td+Tf* + Bis-enoxacin 25 mg	0.0±0.0*	263.5±113.6**	0.0±0.0*	1.0±0.6
*Pg+Td+Tf* + Alendronate 1 mg	171.0±0.0*	265.5±61.9**	1.0±0.0	1.1±1.1
*Pg+Td+Tf* + Alendronate 10 mg	81.9±8.9*	290.9±106.8	3.3±1.5	1.3±0.8
*Pg+Td+Tf* + Enoxacin 5 mg	0.0±0.0	277.5±143.2**	0.0±0.0*	1.0±1.3
*Pg+Td+Tf* + Doxycycline 5 mg	63.3±37.4	389.5±62.4**	2.0±0.0	0.2±0.4*
Sham-infected control	63.4±3.2	327.4±122.3**	1.0±0.0	0.3±0.5*

Histological analysis of rat jaws (H&E staining). Each molar surface was evaluated for apical migration of JE and the length of migration along the cementum was measured in μm. Vertical bone resorption was also examined by measuring the length in μm from the CEJ to ABC. Polymorphonuclear neutrophils and blood vessels along the interdental area were counted in 50 μm^2^ sections. Asterisks indicate the following *P*-values when compared to controls *P<0.05, ** *P*<0.01.

### Systemic spread of bacteria from periodontium to internal organs

Furthermore, the ability of these pathogens to attach, invade junctional epithelial cells, and spread to systemic organs was evaluated and results showed a systemic presence of bacterial genomic DNA from *P. gingivalis, T. denticola* and *T. forsythia. P. gingivalis* and *T. denticola* were found in the heart, aortic arch, thoracic aorta and abdominal aorta of all polymicrobial infected groups regardless of treatment (Table 4). *T. forsythia* was found in the heart, aortic arch, thoracic aorta, and liver of infected groups (Table 4). These data suggest that periodontal pathogens not only colonize the oral cavity but also spread into different internal organs in infected rats, but that none of the therapeutic strategies prevented systemic migration.

**Table 4:** Distribution of organ samples positive for bacterial DNA by PCR.

		*P. gingivalis*						*T. denticola*						*T. forsythia*					
Groups	n	H	A	TA	AA	L	S	H	A	TA	AA	L	S	H	A	TA	AA	L	S
*Pg+Td+Tf*	6	2	0	4	0	1	0	3	1	3	2	0	0	4	0	1	0	3	0
*Pg+Td+Tf* + Bis-enoxacin 5 mg	6	0	0	0	3	0	0	3	1	0	0	0	0	0	1	0	0	0	0
*Pg+Td+Tf* + Bis-enoxacin 25 mg	6	0	2	0	0	0	0	4	3	1	2	0	0	0	3	1	0	0	0
*Pg+Td+Tf* + Alendronate 1 mg	6	0	0	1	1	0	0	3	2	0	0	0	0	0	4	0	0	0	0
*Pg+Td+Tf* + Alendronate 10 mg	6	0	0	0	0	0	0	0	0	0	1	0	0	2	1	0	0	0	0
*Pg+Td+Tf* + Enoxacin 5 mg	6	3	0	0	0	0	0	3	1	0	2	0	0	5	2	0	0	0	0
*Pg+Td+Tf* + Doxycycline 5 mg	6	4	0	0	2	0	0	2	0	0	0	0	0	0	2	1	0	0	0
Sham-infected control	6	0	0	0	0	0	0	0	0	0	0	0	0	0	0	0	0	0	0

Rats euthanized at 12 weeks post initial polymicrobial infection, infection and treatment or mock-infection. Organs (H-Heart, A-Aortic Arch, TA-Thoracic Aorta, AA-Abdominal Aorta, L-Liver, and S-Spleen) were analyzed for the presence of bacterial genomic DNA using appropriate bacteria-specific PCR primers. The number of rats in each group that were positive for bacterial genomic DNA via PCR is shown.

## Discussion

In this article we show for the first time that bis-enoxacin inhibits alveolar bone resorption induced by periodontal bacterial infection. Bis-enoxacin is a new type of anti-resorptive that inhibits binding between the B-subunit of V-ATPase and microfilaments and osteoclast formation and resorption [24]. Enoxacin and bis-enoxacin inhibit osteoclasts by disrupting specialized vesicular trafficking necessary for osteoclast formation and bone resorption [24], [25]. Inhibition included failure of tartrate-resistant acid phosphatase to be proteolytically-activated from its pro-enzyme, a process that normally occurs during vesicle trafficking [35]. Enoxacin and bis-enoxacin altered the plasma membrane distribution of dendritic cell-specific transmembrane protein, which is a cell fusogen required for the formation of multinuclear cells [36]. They also prevented transport of V-ATPase to the ruffled plasma membrane, which is required for bone resorption [37]. Our data suggest that bis-enoxacin may prove to be an effective agent for preventing alveolar bone resorption associated with periodontal infections. Periodontal disease (PD) therapies generally rely on compounds that have antibacterial capabilities to reduce bacterial infection and the consequent inflammation that triggers alveolar bone resorption. In principle, agents that are antibacterial, anti-resorptive, and anti-inflammatory could be particularly effective. However, there are serious concerns regarding using anti-resorptives for therapy due to the association of current anti-resorptives with osteonecrosis of the jaw (ONJ) [16]. Hence, it is vital to find new agents that do not induce osteonecrosis or which, when used in combination with osteonecrosis-inducing anti-resorptives, ameliorate their effects. Although we do not know whether enoxacin or bis-enoxacin are associated with increased ONJ, we do know that they inhibit osteoclasts by a different mechanism from both conventional bisphosphonates and denosumab (another commonly used anti-resorptive also associated with ONJ) [18]. Because of this it is possible they will not have the same link to osteonecrosis.

Enoxacin was developed as an antibiotic in the 1980's [23] and continues to be widely used throughout most of the world although it was withdrawn from the US market. Recently, enoxacin was identified in separate screens of small molecules that block binding between the V-ATPase and microfilaments [17] or stimulate microRNA activity [22]. Enoxacin was shown to inhibit osteoclast bone resorption *in vitro* [17], [25]. It has also been shown to inhibit human cancer growth and metastasis in a xenobiotic mouse model [22]. These data are particularly exciting in that enoxacin has few effects on most cell types and human use has been associated with only relatively minor off-target effects [38], particularly when compared with cancer.

Bis-enoxacin was originally developed as a bone-targeted antibiotic for the treatment of osteolysis [23]. Because enoxacin is an antibiotic, it is difficult to envision its use to treat bone diseases like osteoporosis given the systemic doses that would be required to achieve local concentrations in the bone microenvironment adequate to inhibit osteoclasts. Such doses would generally kill commensal bacteria and risk the evolution of antibiotic-resistant strains. However, bisphosphonates are quickly targeted to bone where they accumulate. Relatively low systemic doses may allow accumulation of therapeutic doses in the bone. We previously showed that bis-enoxacin inhibits osteoclast activity induced aseptically by mechanical force in a rat orthodontic tooth movement model [24]. The combination of a bone targeted anti-resorptive and antibiotic would seem well-suited for the treatment of PD and our data are consistent with that idea.

This study made use of a recently developed polymicrobial model of periodontal disease in rats [27] and mice [29]. Data show that the rats used in this study were able to be successfully colonized with these pathogens for a period of time long enough to cause periodontal disease. In our study we found that bis-enoxacin reduced gingival inflammation, alveolar bone resorption and intrabony defects in a rat periodontal disease model. Reduction of alveolar bone resorption by bis-enoxacin exceeded the reduction induced by alendronate, a more potent anti-resorptive that functions by blocking the mevalonate pathway in osteoclasts [39]. It is possible that the unique property of bis-enoxacin of having both anti-resorptive and antibiotic activities made it particularly useful in blocking alveolar bone resorption triggered by periodontal disease in the current study.

This study represents a first step in evaluating bis-enoxacin as a novel therapeutic agent for clinical use in periodontal disease. It was beyond the scope of our study to discern whether the effects of bis-enoxacin on alveolar bone resorption triggered by periodontal infection are the result of the anti-resorptive or antibiotic activities of bis-enoxacin. We propose that the therapeutic activity of bis-enoxacin is due to a combination of the two. Further work using fluoroquinolone-derivatives of bisphosphonates that retain only their anti-resorptive or antibiotic activities [17], [23], [32], [40] to precisely decipher the therapeutic potential will be required. Two molecular functional activities have been identified in bis-enoxacin that could account for inhibition of osteoclast bone resorption. Previously, we showed that bis-enoxacin blocks a vital binding interaction in osteoclasts between V-ATPase and microfilaments [24]. We believe that the accumulated data best support this mechanism as the cause of the ability of enoxacin and bis-enoxacin to inhibit osteoclasts [18]. Further, recent studies show that enoxacin stimulates microRNA activity, which could have anti-resorptive and/or anti-inflammatory activities [21].

Enoxacin was shown to prevent the growth and metastasis of colorectal and prostate cancers due to stimulation of microRNAs [22], [41]. It will be of interest to test bis-enoxacin to determine whether it also stimulates microRNAs. If bis-enoxacin retains anti-cancer activity, it may prove to be a new agent for the treatment of bone cancer. The current study proposes the use of bis-enoxacin for clinical use against periodontal disease. Bis-enoxacin's ability to reduce alveolar bone resorption following periodontal infection as well as to reduce orthodontic tooth movement [24] makes it a potent candidate for clinical use. Prior to its use in the clinic it will be vital to ensure that bis-enoxacin, unlike other anti-resorptives, does not increase the incidence of osteonecrosis of the jaw.
